# Genetic and epigenetic contributors to cleft laterality: evidence from monozygotic mirror twins and replication cohorts

**DOI:** 10.1093/hmg/ddag088

**Published:** 2026-09-11

**Authors:** Aline L Petrin, Waheed Awotoye, Christina E Spencer, Ligiane A Machado-Paula, Luke Hovey, Henry Keen, Michael Chimenti, Patrick Breheny, Fang Qian, Shareef Dabdoub, James C Thomas, Azeez Butali, Jeffrey C Murray, Shankar Rengasamy Venugopalan, Lina M Moreno-Uribe

**Affiliations:** Department of Orthodontics, College of Dentistry and Dental Clinics, University of Iowa, 801 Newton Rd, Iowa City, IA 52242, United States; Department of Orthodontics, College of Dentistry and Dental Clinics, University of Iowa, 801 Newton Rd, Iowa City, IA 52242, United States; College of Dentistry and Dental Clinics, University of Iowa, 801 Newton Rd, Iowa City, IA 52242, United States; Iowa Institute of Human Genetics, Carver College of Medicine, University of Iowa, 431 Newton Road, 117 EMRB, Iowa City, IA 52242, United States; Department of Orthodontics, Tufts University School of Dental Medicine, 1 Kneeland St, Boston, MA 02111, United States; Iowa Institute of Human Genetics, Carver College of Medicine, University of Iowa, 431 Newton Road, 117 EMRB, Iowa City, IA 52242, United States; Iowa Institute of Human Genetics, Carver College of Medicine, University of Iowa, 431 Newton Road, 117 EMRB, Iowa City, IA 52242, United States; Department of Biostatistics, College of Public Health, University of Iowa, 145 N Riverside Dr, Iowa City, IA 52242, United States; Division of Biostatistics and Computational Biology, College of Dentistry and Dental Clinics, University of Iowa, 801 Newton Rd, Iowa City, IA 52242, United States; Division of Biostatistics and Computational Biology, College of Dentistry and Dental Clinics, University of Iowa, 801 Newton Rd, Iowa City, IA 52242, United States; Division of Biostatistics and Computational Biology, College of Dentistry and Dental Clinics, University of Iowa, 801 Newton Rd, Iowa City, IA 52242, United States; Department of Oral Pathology, Radiology, & Medicine, College of Dentistry and Dental Clinics, University of Iowa, 801 Newton Rd, Iowa City, IA 52242, United States; Department of Pediatrics, Carver College of Medicine, University of Iowa, 200 Hawkins Drive, Iowa City, IA 52242, United States; Department of Orthodontics, Tufts University School of Dental Medicine, 1 Kneeland St, Boston, MA 02111, United States; Department of Orthodontics, College of Dentistry and Dental Clinics, University of Iowa, 801 Newton Rd, Iowa City, IA 52242, United States

**Keywords:** complex disease, epigenetics, DNA methylation, whole genome sequencing, epigenomics, ggenomics

## Abstract

Nonsyndromic cleft lip (nsCL) exhibits a non-random laterality pattern, with left-sided clefts occurring twice as frequently as right-sided clefts. The molecular mechanisms underlying this laterality bias remain poorly understood. We performed whole-genome sequencing and methylation profiling on a family comprising monozygotic twins with mirror-image nsCL, their affected mother, and unaffected father and brother. We conducted three independent replications via publicly available whole genome data; genome-wide methylation analysis in 38 individuals with unilateral cleft; and validation of methylation results in the top 3 candidate genes in 385 unrelated individuals with unilateral nonsyndromic cleft lip with or without cleft palate (nsCL/P) (DNA from blood or saliva). We identified a variant in *FGF20* (p.Ile79Val) shared by the twins and their mother. We observed laterality and severity-associated methylation differences in three main genes. *ARID5B* showed higher methylation in left clefts (saliva, *P* = .001; blood, *P* = .032). *ZFP57* demonstrated a strong cleft-extent effect, with cleft lip and palate (CLP) showing markedly higher methylation than cleft lip only (CL) (LCLP vs. RCL p^adj^ = 0.0004; LCLP vs. LCL p^adj^ = 0.019). *HOOK2* displayed a cross-tissue cleft-extent effect in the opposite direction—CLP subtypes were hypomethylated relative to CL-only subtypes in blood (*P* < .0001) and saliva (*P* = .0008). This study provides evidence that DNA methylation patterns play a role in both the laterality and severity of cleft lip. *ARID5B* provides a consistent laterality signal across tissues, while *ZFP57* and *HOOK2* track palatal involvement independently of side. Together, these findings suggest that epigenetic variation acts downstream of genetic predisposition to shape cleft phenotypes.

## Introduction

Orofacial clefts (OFCs) are among the most common congenital malformations, affecting approximately 1 in 700 live births worldwide [1]. These conditions result from disturbances during embryonic development that lead to incomplete fusion of the upper lip (cleft lip), palate (cleft palate), or both [2]. Approximately 70% of cases are classified as nonsyndromic, occurring without additional congenital anomalies [3]. OFCs impose significant functional, aesthetic, and psychosocial burdens on affected individuals, requiring multidisciplinary care from infancy through adulthood.

A striking epidemiological feature of unilateral cleft lip is its non-random laterality distribution with left-sided clefts occurring approximately twice as frequently as right-sided clefts, a pattern consistently observed across diverse populations and geographic regions [1]. This 2:1 ratio strongly suggests that cleft laterality is not a random developmental event but reflects underlying biological mechanisms that preferentially affect left-sided craniofacial development. Despite decades of research, the molecular basis of this laterality bias remains largely unexplained.

Monozygotic twins provide an invaluable model for studying the contribution of genetic and environmental factors to birth defects, as they share nearly identical genetic backgrounds [4]. Mirror-image traits, occurring in approximately 25% of monozygotic twin pairs, include hair whorls, birthmarks, and structural anomalies expressed on opposite sides of the body midline [5]. Monozygotic twins with mirror-image unilateral clefts—where one twin has a left-sided cleft and the other a right-sided cleft—represent a particularly powerful natural experiment for studying laterality determination while controlling for genetic background.

Previous whole-genome sequencing studies of mirror twins with OFCs found no discordant genetic variants that could explain the laterality differences, suggesting that epigenetic mechanisms may play a crucial role in modifying cleft laterality [6]. DNA methylation, a key epigenetic modification involving the addition of methyl groups to cytosine residues at CpG sites, has emerged as a critical factor in OFC pathogenesis [7–10]. By affecting transcription factor binding at gene regulatory regions, DNA methylation can modulate gene expression during critical developmental windows [11, 12].

Genetic studies of nonsyndromic OFCs have identified multiple susceptibility loci, implicating genes such as *IRF6, PAX7, MAFB, ABCA4*, and *VAX1*, among others [13]. While cleft lip (CL) and cleft lip and palate (CLP) share many of these risk loci, they show partially distinct genetic profiles consistent with separable developmental processes for lip fusion and palatal fusion. By contrast, far less is known about the genetic determinants of cleft laterality. The limited studies examining left- versus right-sided clefts have not identified consistently replicated variants that specifically determine cleft side [14, 15], suggesting that laterality may be influenced by developmental stochasticity, environmental asymmetry, or epigenetic variation established during embryogenesis. Furthermore, while the clinical distinction between laterality (left vs. right) and cleft extent (whether the palate is involved) is well established, these axes of phenotypic heterogeneity are rarely analyzed separately in epigenetic studies. Cleft lip results from failure of fusion of the maxillary and nasal processes at weeks 4–7 of gestation, whereas palatal clefting involves secondary palate fusion at weeks 8–12 [2]. This temporal separation suggests that laterality and cleft extent may have distinct molecular underpinnings that warrant independent investigation.

In this study, we present a comprehensive multi-omic analysis of a female monozygotic twin pair with mirror-image nonsyndromic cleft lip, born to a mother with left-sided microform cleft lip. By integrating whole-genome sequencing with genome-wide DNA methylation profiling and conducting three independent replications, we identify candidate molecular mechanisms that may contribute to both the occurrence and the laterality bias of this common birth defect.

## Results

### Whole-genome sequencing identifies shared protein-disrupting variants

Analysis of discordant protein-disrupting variants between the twins identified a heterozygous variant in *KRT6B* present only in the twin with left cleft lip (LCL). Although monozygotic twins share a common zygote, post-zygotic somatic mutations can arise independently in each twin after embryonic splitting, resulting in variants detectable in only one twin [16]. The *KRT6B* variant may represent such an event; however, its presence in the unaffected father and heterozygous state made it unlikely to explain the laterality difference. A de novo splice site variant in *ACLY* was also identified. While no evidence suggests a role for this gene in craniofacial development, the knockout mouse showed early embryonic (stage E7) lethality. Additionally, we identified a protein-disrupting variant of uncertain significance in *MUC3A* that was maternally-inherited (heterozygous). Based on the in-silico prediction and the likely recessive inheritance pattern, we concluded that this heterozygous maternally-transmitted variant was unlikely to be the risk variant.

Consistent with previous reports [14], we did not identify discordant pathogenic variants that could definitively explain the laterality differences. We therefore focused on maternally transmitted variants shared by both twins and their affected mother (Table 1). This approach identified 16 rare protein-disrupting variants in craniofacial candidate genes (Table 2).

**Table 1:** Number of variants (CADD > 15) at each analytical step towards prioritization of pathogenic variants.

Filter	Number of Variants
Protein-disrupting variants with MAF < 1%	955 (MZ-1); 939 (MZ-2)
Maternally inherited protein-disrupting variants with MAF < 1%	393 (MZ-1); 416 (MZ-2)
Shared maternally inherited protein-disrupting variants with MAF < 1%	387
Variants with CADD≥15	175
Variants with CADD≥15 + deleterious (SIFT)	90
Variants with CADD≥15 + damaging (Polyphen2)	82
Variants with CADD≥15 + deleterious (SIFT) + damaging (Polyphen2)	59
Variants with CADD≥15 in Craniofacial genes	16
Variants with CADD≥15 + deleterious (SIFT) and/or damaging (Polyphen2) in craniofacial genes	7

Note: Rows 5–7 (CADD≥15 + SIFT; CADD≥15 + PolyPhen2; CADD≥15 + both) represent parallel independent filters applied to row 4, not nested sequential subsets of each other. Row 8 applies all three conditions simultaneously to the craniofacial gene list.

**Table 2:** Maternally-transmitted shared variants with evidence supporting the roles of the genes in craniofacial development.

#	Chrom:Pos	Ref/Alt	Gene	HGVS p.	SIFT	PolyPhen	CADD	Facial phenotype	Craniofacial phenotype	Cleft genes (DB)	ACMG cat	Inheritance
1	7:7452365	G/C	*COL28A1*	NP_001032852.2: p.Pro488Arg	tolerated	Possibly damaging	22.9	Mid forehead	Nil	Nil	−13 (B)	LR
2	9:126503192	C/T	*MVB12B*	NP_258257.1: p.Arg297Cys	tolerated	Probably damaging	26.4	Self-reported chin dimples	Nil	Nil	0 (VUS)	LR
3	5:113028959	C/T	*MCC*	NP_001078846.2: p.Val952Met	deleterious	Probably damaging	23.8	Upper lip center	Nil	Nil	0 (VUS)	VLR
4	13:37590506	C/T	*POSTN*	NP_006466.2: p.Gly103Ser	tolerated	Probably damaging	24.9	Nil	Abnormal ameloblast morphology; Abnormal tooth eruption	Nil	0 (VUS)	VLR
5	21:40296147	C/T	*DSCAM*	NP_001380.2: p.Arg697Gln	tolerated	benign	20.9	Nil	Abnormal cranial cavity morphology; dome cranium; small ears	Nil	−11 (B)	VLD
6	4:2833015	C/G	*SH3BP2*	NP_003014.3: p.Ser505Cys	tolerated	benign	15.58	Nil	Abnormal craniofacial bone morphology	Nil	−14 (B)	VLR
7	15:89291615	A/C	*FANCI*	NP_001106849.1: p.Leu631Phe	tolerated	benign	15.43	Nil	Abnormal craniofacial bone morphology; Cleft palate	Nil	−21 (B)	VLR
8	20:51790193	A/G	*SALL4*	NP_065169.1: p.Ser764Pro	tolerated	benign	21	Nil	Abnormal craniofacial bone morphology; Cleft palate	Nil	−21 (B)	VLD
9	16:2100458	C/T	*PKD1*	NP_001009944.3: p.Arg3169Gln	tolerated	benign	16.98	Nil	Abnormal craniofacial bone morphology; short maxilla	Nil	−2 (LB)	VLD
10	16:81044059	G/A	*ATMIN*	NP_056066.2: p.Val521Ile	tolerated	benign	21.6	Nil	Abnormal craniofacial morphology; micrognathia; Thick upper lip	Nil	−12 (B)	VLR
11	5:84064787	T/C	*EDIL3*	NP_005702.3: p.Ile289Val	tolerated	benign	22.7	Nil	Abnormal pinna cartilage morphology; floppy ears	Nil	−5 (LB)	LR
12	8:17001798	T/C	*FGF20*	NP_062825.1: p.Ile79Val	deleterious	Possibly damaging	26.8	Nil	Abnormal tooth morphology	Nil	−15 (B)	VLD
13	8:117835473	C/T	*EXT1*	NP_000118.2: p.Val379Ile	tolerated	benign	16.39	Nil	Cleft of Secondary palate	Yes	0(VUS)	VLD
14	5:37180175	G/A	*CPLANE1*	NP_075561.3: p.Pro1860Leu	deleterious	benign	22.6	Nil	Cleft of upper lip; Cleft palate (Joubert syndrome)	Yes	−7 (B)	VLR
15	14:39180971	C/T	*PNN*	NP_002678.3: p.Ala421Val	tolerated	benign	17.38	Nil	Cleft palate	Yes	−3 (LB)	VLR
16	15:74722964	C/T	*CYP1A1*	NP_001306146.1: p.Gly45Asp	deleterious	Possibly damaging	22.8	Nil	Nil	Yes	−14 (B)	LR

Facial phenotype annotations are from the Facial Morphology GWAS [17]. Craniofacial phenotype annotations are derived from the Mouse Genome Informatics database (MGI; www.informatics.jax.org) and the Online Mendelian Inheritance in Man database (OMIM; www.omim.org). Cleft gene database (DB) annotations are from CleftGeneDB [18]. ACMG categories: B = Benign; LB = Likely Benign; VUS = Variant of Uncertain Significance. Inheritance: LR = Low Recessive; VLR = Very Low Recessive; VLD = Very Low Dominant. Nil = no annotation found in the respective database.

These variants were novel, or rare in population control databases, altered protein sequences, and ranked among the top 1% most deleterious mutations in the human genome (based on CADD scores ≥15). Several of these variants were in genes known to be associated with craniofacial development based on evidence from facial gene scan databases [17], mouse genome informatics, and cleft gene databases. Notably, variants in *FANCI, SALL4, EXT1, CPLANE1*, and *PNN* were identified in genes that, when manipulated in mice, resulted in cleft phenotypes (CleftGeneDB; https://bioinfo.uth.edu/CleftGeneDB) [18].

Table 2 shows mutations that have been considered deleterious, possibly damaging, or probably damaging by at least one prediction tool (*COL28A1, CPLANE1, CYP1A1, MCC, POSTN, FGF20*, and *MVB12B*). The mutations in *COL28A1, CPLANE1, CYP1A1* and *MVB12B* were shared by the twins, the mother and the unaffected brother. The mutations in *MCC, POSTN* and *FGF20* were shared by the three affected individuals (twin girls and mother) and not present in the unaffected father and brother.

Of these potentially pathogenic protein-disrupting variants in craniofacial genes, only the *FGF20* variant (p.Ile79Val) was predicted by DOMINO to follow a dominant inheritance pattern; thus, becoming our main candidate gene.

Protein function analysis showed that the affected isoleucine residue resides within the fibroblast growth factor family domain, which is critical for receptor binding [19]. Analysis of human craniofacial epigenomics data revealed regulatory element signatures within *FGF20*, with the highest enhancer peaks observed during Carnegie stages 13–17, corresponding to critical timepoints for primary palate formation [20]. Significant *FGF20* expression appeared in craniofacial tissues from CS13 through CS22 (*P* < .05), supporting its role in lip and palate development [21].

Our replication analysis with publicly available whole-genome sequencing data from multiethnic trios affected with nonsyndromic cleft lip and palate [22] did not show significant results; however previous genome-wide association study investigating sex as a modifier of variant risk identified a novel cleft risk locus at chromosome 8p22, which is within a topologically associating domain (TAD) containing *FGF20.* That study also demonstrated robust *Fgf20* expression in the developing mouse maxilla between days E10.5 and E13.5 [23].

### In silico analyses reveal significant FGF20 transcription in craniofacial tissues during embryonic development.

To further investigate the role of *FGF20* in human craniofacial development, we examined craniofacial regulatory elements signatures within *FGF20* gene and its spatiotemporal expression during embryonic development. Using data from the expanded encyclopedia of DNA elements (ENCODE) (https://www.encodeproject.org), we identified gene regulatory elements signatures within *FGF20* including enhancer-like, promoter-like and CTCF- binding elements in the first exon and intron of the gene. We further analyzed the Human Craniofacial Epigenomics dataset (GSE97752) from the Cotney Lab (https://www.chopcranio.org/human-craniofacial-development) [20] and confirmed the presence of regulatory element signatures within *FGF20,* revealing craniofacial-specific regulatory elements, evidenced by enhancer peaks within the gene. The highest peaks were observed in developing craniofacial tissue structures between Carnegie stages (CS) 13–17, corresponding to approximately 4–6 weeks of embryonic development which are key timepoints for primary palate formation.

To assess *FGF20* expression in human craniofacial tissues during embryogenesis, we analyzed bulk and single-cell RNA sequencing (scRNA-seq) data from human craniofacial tissues collected between 4–8 weeks of gestation (GSE197513, phs002008) [21]. Significant *FGF20* expression (*P* < .05) appeared in craniofacial tissue structures at CS13 (*P* = .001) and remained elevated through CS14 (*P* = .007), CS15 (*P* = .00096), and CS17 (*P* = .01), at fold changes > 30, > 20, > 30, and > 14, respectively (Fig. 1). This spatiotemporal expression pattern correlates with critical stages of craniofacial tissue development coincident with the timing of palatogenesis, thus supporting a role for *FGF20* in lip and palate development.

**Figure 1:**
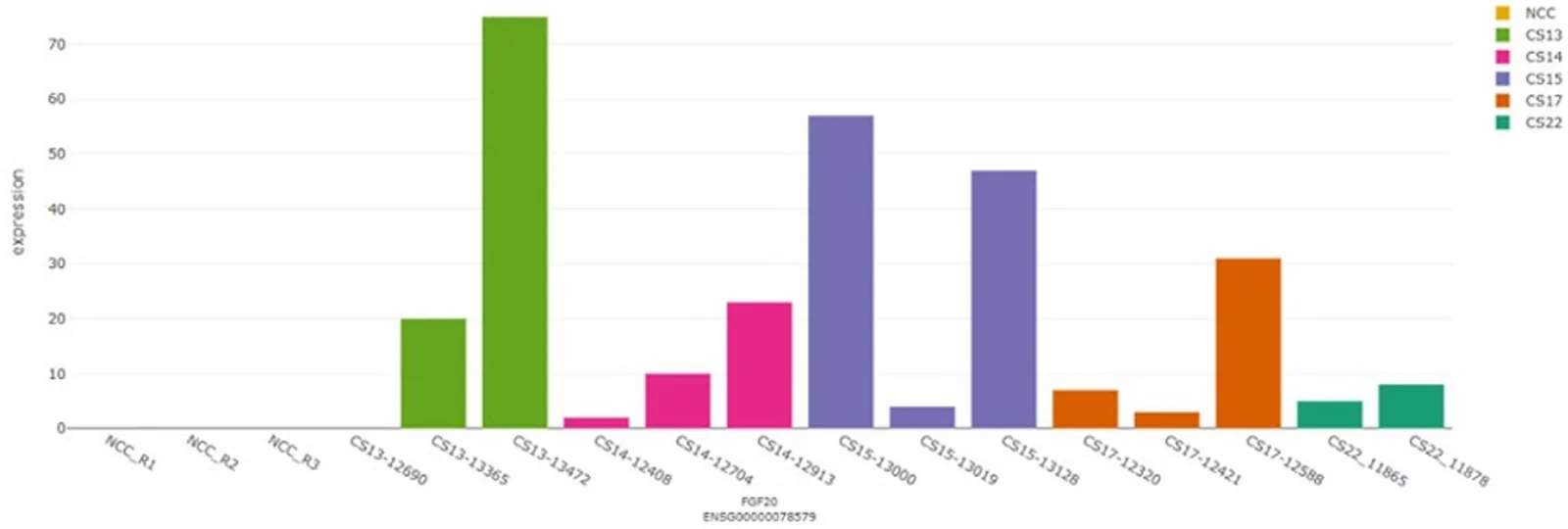
FGF20 expression plot from bulk and single-cell RNA seq of craniofacial tissue between 4–8 weeks post conception. ^*^NCC: Neural crest cells; CS: Carnegie stage.

### DNA methylation analysis identifies differentially methylated regions

Comparison of genome-wide methylation in blood-derived DNA between the mirror twins identified 408 CpG sites with absolute Δβ > 5% (Supplementary Table S1). Of these, 100 showed higher methylation in the twin with LCL, while 308 showed higher methylation in the twin with right cleft lip (RCL). Enrichment analysis of differentially methylated positions (DMPs) within 5 kb of gene transcription start sites revealed significant enrichment for the biological process ‘DNA methylation involved in embryo development’ (FDR p^2^adj = 1.32 × 10^−13^).

Of the 408 DMPs, 40.2% were located in gene body regions, 35.8% in intergenic regions, 11.8% in 5’UTR regions, and the remainder in TSS-proximal or 3’UTR regions. The three candidate genes selected for targeted replication occupy distinct genomic contexts: the *ZFP57*-associated cluster lies entirely in an intergenic region 3.3–4.2 kb upstream of the TSS; the *ARID5B* site (cg22368847) is located in the gene body on chromosome 10; and the *HOOK2* site (cg11738485) is located in the gene body on chromosome 19.

A notable finding was a cluster of 14 differentially methylated CpG sites spanning approximately 0.9 kb (867 bp) in the intergenic region 3295–4162 bp upstream of the *ZFP57* transcription start site on chromosome 6 (hg19: chr6:29648225–29 649 092). All 14 sites showed consistently higher methylation in the twin with LCL (Δβ = 7%–12%), providing convergent evidence for a coordinated regulatory change at this locus rather than an isolated stochastic difference. The site with the lowest Δβ in this cluster (cg06032337) showed borderline replication in an independent cohort, and the adjacent site cg20228636 (11 bp away) was selected as the targeted replication site for the large-scale replication study. By contrast, ARID5B and HOOK2 were each represented by a single DMP, located in gene body regions on chromosomes 10 and 19, respectively.

We conducted a replication study of the 408 CpG sites that showed a Δβ ≥ 5% in a small independent cohort. This cohort consisted of individuals with unilateral nonsyndromic CL/P, which were separated based on laterality of the cleft (right: RCL/P or left: LCL/P) and based on source of DNA (Blood or Saliva). Our analysis revealed a borderline significant CpG site in ZFP57 (saliva, *P* = .06) and significant CpG sites in *ARID5B* (blood, *P* = .002) and *HOOK2* (blood and saliva, *P* = .02).

### Additional replication confirms laterality and cleft extent-associated methylation patterns

To further investigate these associations (*ZFP57, HOOK2 and ARID5B*), we performed a larger-scale tissue-stratified analysis with an independent replication cohort (n = 385), which revealed that methylation differences among cleft subtypes operate along two distinct biological dimensions: cleft laterality (left vs. right side) and cleft extent (CLP vs. CL). Each of the three candidate genes displayed a unique combination of tissue-specific laterality and extent signatures (Tables 3 and 4).

**Table 3:** DNA methylation levels by cleft laterality in the replication cohort (n = 385).

Gene	Tissue	n	Left Mean (%)	Right Mean (%)	Δ Mean (%)	*P*-value
*ARID5B*	Blood	198	70.7	66.5	+4.2	**.032**
*ARID5B*	Saliva	179	62.1	50.5	+11.6	**.001**
*ZFP57*	Blood	198	NS	NS	—	NS
*ZFP57*	Saliva	187	52.3	63.5	−11.1	**.016**
*HOOK2*	Blood	196	NS	NS	—	NS
*HOOK2*	Saliva	187	NS	NS	—	NS

*NS = not significant. Δ Methylation shows direction relative to left clefts.*

**Table 4:** Results for four cleft subtypes and comparisons by gene and tissue.

Gene	Tissue	n	*P*-value	Median methylation by subtype & significant post-hoc pairs
*ZFP57*	Blood	198	**.0003**	LCLP = 56.8%, RCLP = 46.1%, LCL = 31.0%, RCL = 25.4% | Sig. pairs: LCLP>RCL (p adj = 0.0004); LCLP>LCL (p adj = 0.019)
*ZFP57*	Saliva	187	.089	LCLP = 40.5%, RCLP = 56.2%, LCL = 32.9%, RCL = 41.4% | No significant pairs
*HOOK2*	Blood	196	**<.0001**	LCLP = 15.4%, RCLP = 18.1%, LCL = 19.5%, RCL = 20.5% | Sig. pairs: LCLP<RCL (p adj = 0.0003); RCLP<RCL (p adj = 0.002); LCLP<LCL (p adj = 0.012)
*HOOK2*	Saliva	187	**.0008**	LCLP = 14.5%, RCLP = 19.8%, LCL = 19.1%, RCL = 18.5% | Sig. pairs: LCLP<LCL (p adj = 0.001); LCL > RCL (p adj = 0.046)
*ARID5B*	Blood	198	.189	LCLP = 72.5%, RCLP = 65.5%, LCL = 70.7%, RCL = 69.0% | No significant pairs
*ARID5B*	Saliva	179	**.006**	LCLP = 60.5%, RCLP = 54.5%, LCL = 70.9%, RCL = 44.5% | Sig. pairs: LCL > RCL (p adj = 0.015); LCL > RCLP (p adj = 0.024)

LCL-left cleft lip; RCL-right cleft lip; LCLP-left cleft lip and palate; RCLP-right cleft lip and palate

**ARID5B:** The most striking finding emerged from analysis of *ARID5B* (cg22368847), which showed consistently higher methylation in left-sided clefts across both tissue types, representing a cross-tissue, directionally concordant laterality signal. In saliva, left-sided clefts demonstrated mean methylation of 62.1% compared to 50.5% in right clefts (Δ = +11.6%, *P* = .001). This laterality pattern was reproduced in blood-derived samples (left: 70.7%, right: 66.5%; Δ = +4.2%; *P* = .032), with a smaller but statistically significant effect size consistent with the greater homogeneity of blood-derived methylation (Table 3). In saliva, the four cleft subtypes (H = 12.55, *P* = .006) revealed that left cleft lip (LCL) showed the highest median methylation (70.9%), significantly exceeding both right cleft lip (RCL; 44.5%; p^adj^ = 0.015) and right cleft lip and palate (RCLP; 54.5%; p^adj^ = 0.024) (Table 4). The LCL vs. RCL comparison in saliva (raw *P* = .003) represents a laterality-within-CL subtype effect, indicating that the *ARID5B* laterality signal is particularly pronounced in isolated cleft lip cases.

Analysis of human embryonic craniofacial transcriptome data confirmed significant *ARID5B* expression during Carnegie stages 13–22, the precise window of palatal development [21].

**ZFP57:** Analysis of *ZFP57* (cg20228636) revealed a more complex, tissue-divergent pattern. In saliva-derived DNA, right-sided clefts showed significantly higher methylation than left-sided clefts (right: 63.5%, left: 52.3%; Δ = −11.1%; U = 3488, *P* = .016)—the opposite direction from *ARID5B*. This directional contrast between genes is notable: these findings raise the possibility that CpG sites associated with the genomic imprinting machinery (*ZFP57*) may respond differently to asymmetric craniofacial developmental signals than those associated with chromatin remodeling factors (*ARID5B*).

In blood-derived DNA, *ZFP57* showed no significant laterality effect (U = 5432, *P* = .182). Instead, blood revealed a strong and highly significant cleft-extent effect across the four subtypes. CLP subtypes showed markedly higher methylation than CL-only subtypes: median methylation in left cleft lip and palate (LCLP; 56.8%) was significantly higher than in both LCL (31.0%; p^adj^ = 0.019) and RCL (25.4%; p^adj^ = 0.0004) (Table 4). This cleft-extent effect—independent of laterality—suggests that *ZFP57* methylation in blood DNA may reflect the additional epigenetic reprogramming events associated with palatal involvement in CLP, consistent with its role as a master regulator of genomic imprinting at developmental control regions [24–26].

**HOOK2:** Analysis of *HOOK2* (cg11738485) revealed no significant laterality effect in either tissue (blood: *P* = .282; saliva: *P* = .491). Instead, *HOOK2* displayed highly significant cleft-extent effects in *both* types of tissue, the only gene to show cross-tissue consistency for the cleft-extent dimension.

In blood (*P* < .0001), we observed that CLP subtypes were consistently *hypomethylated* relative to CL-only subtypes—a direction opposite to *ZFP57*. LCLP (median = 15.4%) was significantly lower than both RCL (median = 20.5%; p^adj^ = 0.0003) and LCL (19.5%; p^adj^ = 0.012). RCLP (18.1%) was also significantly lower than RCL (p^adj^ = 0.002) (Table 4).

In saliva, we confirmed that LCLP (median = 14.5%) showed significantly lower methylation than LCL (19.1%; p^adj^ = 0.001). Additionally, a saliva-specific within-CL laterality effect emerged: LCL (mean = 32.8%) showed significantly different methylation from RCL (26.5%; p^adj^ = 0.046), driven by a subset of high-methylation outliers in the LCL group (Table 4).

## Discussion

By leveraging the unique power of monozygotic twins with mirror-image clefts and conducting independent replications—an unbiased genome-wide screen in 38 individuals identifying *ARID5B, ZFP57*, and *HOOK2* as the top candidate loci, followed by a tissue-stratified validation cohort of up to 385 individuals—we have identified gene-specific, tissue-dependent epigenetic signatures that distinguish laterality effects from cleft-severity effects at single-CpG resolution.

Our whole-genome sequencing analysis identified a potentially pathogenic variant in *FGF20* (p.Ile79Val) shared by both twins and their affected mother. Previous studies demonstrated co-expression of *FGF20* and *FGFR2* in developing mouse palatal epithelium and our analysis confirmed significant *FGF20* expression in human craniofacial tissues during critical developmental windows. While this variant may contribute to cleft susceptibility, it cannot explain the laterality difference since both twins carry the same mutation.

The identification of three genes—*ARID5B, ZFP57*, and *HOOK2*—with reproducible laterality-associated methylation patterns represents a significant advance in understanding cleft etiology. Critically, tissue-stratified analyses reveal that these genes do not simply reflect a single epigenetic dimension but instead capture two distinct biological signals: cleft *laterality* (which side the cleft occurs on) and cleft *extent* (whether the palate is involved). Understanding this distinction is essential for interpreting the epigenetic architecture of CL/P and for designing future biomarker studies.

***ARID5B*** emerged as the most robustly associated gene across all analyses, showing consistent laterality effects in both blood and saliva—the only gene to do so. The higher methylation in left-sided clefts was particularly pronounced in saliva-derived DNA and in isolated cleft lip cases. As a chromatin remodeling factor and histone demethylase co-activator that regulates transcriptional programs during development [27], *ARID5B* may influence the epigenetic landscape during the asymmetric cellular processes underlying facial development. Its significant expression during Carnegie stages 13–22 positions it precisely at the temporal window when laterality determination and palatal fusion occur [21]. The consistency of the *ARID5B* laterality signal across blood and oral epithelial tissues suggests it may reflect a systemic epigenetic asymmetry established during early embryonic patterning rather than a tissue-specific regulatory response.

***ZFP57*** is particularly notable as a master regulator of genomic imprinting that maintains methylation patterns at imprinting control regions during early development [24, 26]. Its tissue-divergent profile—right-over-left laterality effect in saliva, and CLP-over-CL extent effect in blood—reveals two distinct regulatory layers at this locus. The saliva-based laterality finding (right>left; Δ = −11.1%, *P* = .016) is the opposite in direction from *ARID5B*, consistent with the possibility that imprinting-associated and chromatin remodeling-associated methylation may respond differently to the same asymmetric developmental signals. The blood-based cleft-extent effect indicates that palatal involvement is associated with substantially higher *ZFP57* methylation in circulating DNA. Because *ZFP57* regulates imprinting at multiple genomic loci, its differential methylation may have broad downstream effects on imprinted gene expression during craniofacial morphogenesis. Protein interaction analysis confirmed that *ZFP57* and *TFAP2A* participate in networks associated with left–right axis specification, providing a potential mechanistic link between our epigenetic findings and laterality determination [28].

***HOOK2*** displayed the strongest and most consistent cleft-extent epigenetic signal of the three genes, with CLP subtypes significantly hypomethylated relative to CL-only subtypes in both blood and saliva. The LCLP vs. RCL comparison in blood represents the single most statistically significant post-hoc result in the entire replication study. This CLP hypomethylation direction is strikingly opposite to ZFP57, raising the possibility that methylation at loci involved in distinct molecular pathways—imprinting maintenance and cytoskeletal gene regulation—may be differentially regulated in the context of palatal involvement. HOOK2 encodes a microtubule-binding protein critical for centrosome positioning, Golgi ribbon organization, and polarized cell migration [29]. During palate development, the mesenchymal cells of the palatal shelves must coordinate cytoskeletal remodeling for shelf elevation and horizontal reorientation—processes that depend heavily on microtubule dynamics and cell polarity [29]. Lower HOOK2 methylation in CLP patients may correspond to altered expression states in these cytoskeletal programs during palatal morphogenesis. Additionally, a saliva-specific LCL vs. RCL difference suggests that HOOK2 oral epithelial methylation captures an additional laterality-within-CL signal, albeit of borderline significance.

The tissue-specific divergence of epigenetic signals across all three genes underscores a critical methodological consideration for future epigenetic studies of CL/P. Blood and saliva-derived DNA capture fundamentally different aspects of the methylation landscape: blood-derived methylation reflects hematopoietic and systemic developmental programming, while salivary methylation may more directly capture the epigenetic state of craniofacial epithelial progenitors. Consistent with this interpretation, Substantial variation in DNA methylation profiles across purified blood cell lineages has been demonstrated, and these tissue-level differences are further amplified in comparisons between blood and mucosal tissues [30, 31]. In our data, *ZFP57* exemplifies this divergence most starkly: it shows a laterality effect only in saliva and a cleft-extent effect only in blood, suggesting that distinct regulatory mechanisms operate in parallel compartments. By contrast, *ARID5B*’s laterality signal and *HOOK2*’s extent signal are more broadly shared, consistent with their roles in foundational chromatin and cytoskeletal programs that may be active across multiple tissue lineages.

Our results reveal two distinct epigenetic dimensions in the biology of unilateral CL/P that should be distinguished in future studies: cleft laterality and cleft extent. These dimensions appear to be regulated by different genes and different tissues, and they operate independently. *ARID5B* methylation is primarily a laterality marker; *ZFP57* (blood) and *HOOK2* (both tissues) are primarily cleft-extent markers; and *ZFP57* (saliva) and *HOOK2* (saliva, CL group) bridge both dimensions. This multi-dimensional architecture parallels the known genetic architecture of OFC, in which CLP and CL have partially distinct genetic etiologies and risk loci [2, 8]. The epigenetic findings reported here suggest that these genetically distinct developmental programs are also epigenetically distinguishable at specific CpG sites.

The stronger laterality effects observed in isolated cleft lip cases for *ARID5B* and *HOOK2* may reflect differential sensitivity of epigenetic regulation across developmental stages. Cleft lip results from failure of fusion of the maxillary and nasal processes during weeks 4–7 of gestation, while palatal clefting involves secondary palate fusion during weeks 8–12 [2]. The presence of palatal involvement (CLP) may introduce additional epigenetic heterogeneity that dilutes the pure laterality signal, explaining why laterality-specific methylation differences are more pronounced in CL-only cases.

A notable demographic difference between the blood and saliva groups in our replication cohort (blood: 92.9% non-white; saliva: 57.5%–59.9% non-White across genes) warrants acknowledgment. This population composition difference may contribute to some of the observed tissue divergences and should be explored in future population-stratified analyses. Nonetheless, the directional consistency of findings within each tissue group, and the concordance between our discovery data in the mirror twins and the replication results for *ARID5B* and *ZFP57* laterality effects, support the biological reproducibility of these signals.

Beyond the three candidate genes investigated in the large-scale replication cohort, several additional CpG sites showed suggestive associations in the replication 1 screen that warrant future investigation. Notably, cg27106643, located in the gene body of *NFATC1* (Nuclear Factor of Activated T-cells C1; combined *P* = .037, blood *P* = .007), is embedded within a gene that encodes a transcription factor with established roles in osteoclast differentiation, bone remodeling, and craniofacial morphogenesis, processes directly relevant to palatal and lip development [32]. Similarly, cg25952596, located near the *RTL1/DIO3* imprinted locus on chromosome 14q32 (combined *P* = .025, blood *P* = .005), belongs to a major genomic imprinting domain, extending the imprinting-related methylation theme identified at *ZFP57* in our discovery cohort [33, 34]. Additionally, cg15256244, near *SHISA5*—an antagonist of *WNT* and *FGF* signaling pathways central to palatogenesis—showed a strong blood-specific signal (*P* = .004) consistent with the tissue-specific patterns observed for *ARID5B* [35]. These findings should be interpreted cautiously given the exploratory nature of the replication 1 screen, the number of sites tested, and the absence of large-scale validation. Nevertheless, they represent biologically plausible candidates for independent replication in future epigenome-wide association studies of orofacial clefts.

From a translational perspective, our findings establish the groundwork for developing epigenetic biomarkers for cleft risk assessment and phenotype prediction. *ARID5B* methylation, as the most consistent cross-tissue laterality marker, may have the greatest potential as an accessible biomarker from blood or saliva. The reversible nature of DNA methylation also opens possibilities for preventive interventions, as methylation patterns can be influenced by nutritional factors affecting one-carbon metabolism, including folate, methionine, and choline [12].

Our study has limitations. The discovery analysis was limited to a single twin pair, inherent to the rarity of mirror-image clefts. However, our two-stage replication strategy—an unbiased genome-wide screen followed by a targeted tissue-stratified validation in up to 385 individuals across multiple population groups—substantially strengthens our findings. Population composition differences between tissue groups limit direct cross-tissue comparisons. Future studies should incorporate matched tissue collection (blood and saliva from the same individuals), population-stratified analyses, and mechanistic studies linking the identified CpG sites to gene expression during craniofacial morphogenesis.

## Materials and methods

### Mirror twins and family members

The discovery cohort consisted of a family with female monozygotic twins, their unaffected brother, and their parents. One twin presented with left-sided microform cleft lip (LCL), while the other exhibited right-sided microform cleft lip (RCL). The twins’ mother had a left-sided microform cleft lip; the brother and father had no structural abnormalities (Fig. 2). Blood samples were collected from all available family members. The study was approved by the University of Iowa Institutional Review Board (protocols 199 804 081, 200 003 065, and 200 710 721), and all participants (discovery and replication cohorts) provided written informed consent.

**Figure 2:**
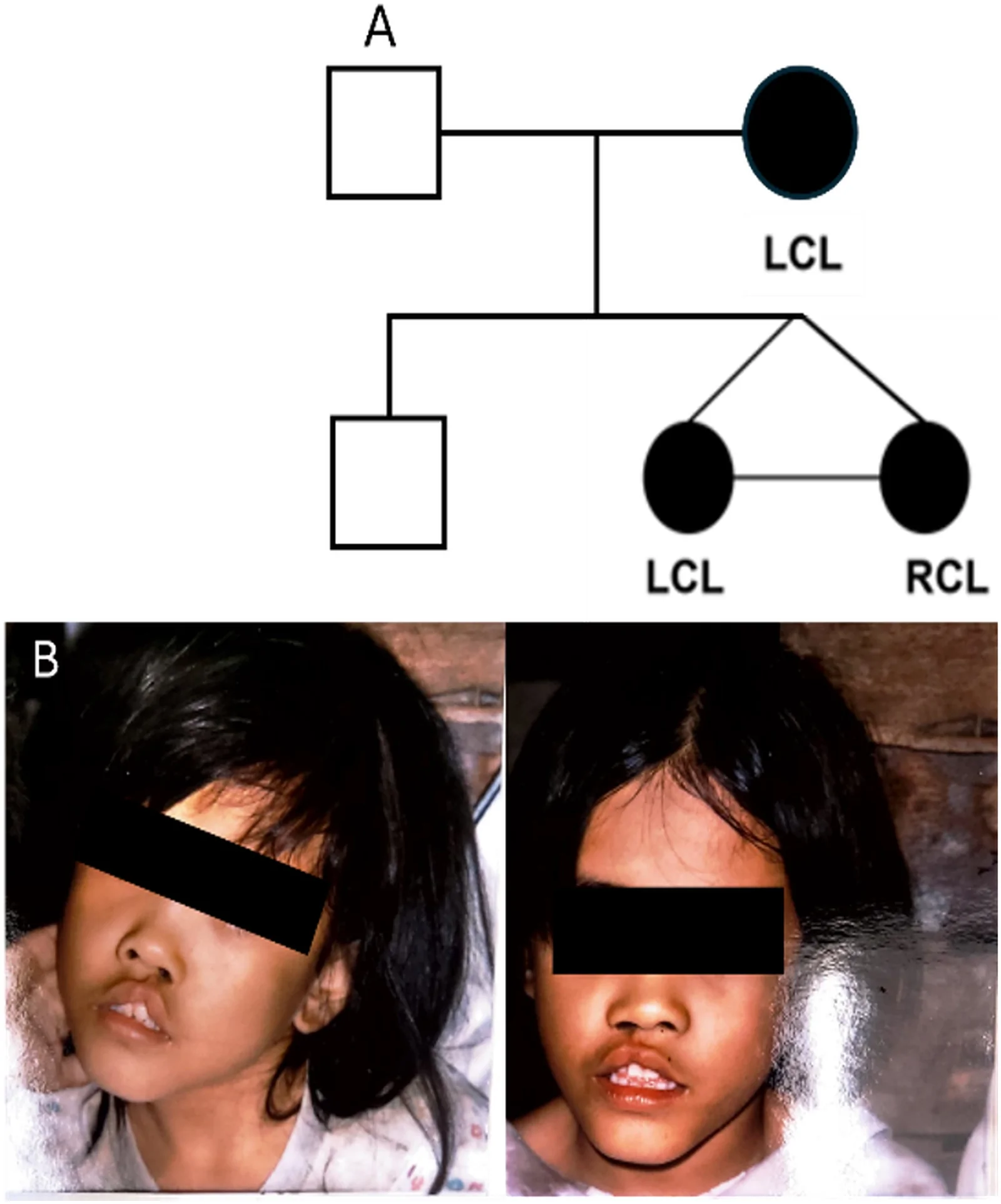
(A) Pedigree showing the inheritance pattern of clefting in the family. (B) Monozygotic twins with mirror cleft lip.

### Whole-genome sequencing

Whole-genome sequencing was performed at an average coverage depth of 30×. Sequence data were aligned to the human genome assembly GRCh38 using the Dynamic Read Analysis for GENomics (DRAGEN) pipeline. We prioritized high-confidence variants (genotype quality ≥ 20, read depth ≥ 10) that were rare or novel (minor allele frequency < 1% in gnomAD). Protein-disrupting variants—defined as missense variants and predicted loss-of-function variants (stop-gained, frameshift, and canonical splice-site variants within ±2 bp of exon-intron junctions)—were assessed using SIFT, PolyPhen2, and CADD. Loss-of-function consequences were assigned based on Ensembl Variant Effect Predictor (VEP) annotations; we use the term ‘protein-disrupting’ throughout to encompass these variant classes. Variants were classified according to the American College of Medical Genetics and Genomics (ACMG) system, and the DOMINO algorithm was used to assess dominant inheritance patterns [36]. Our workflow is illustrated and detailed in Fig. 3.

**Figure 3:**
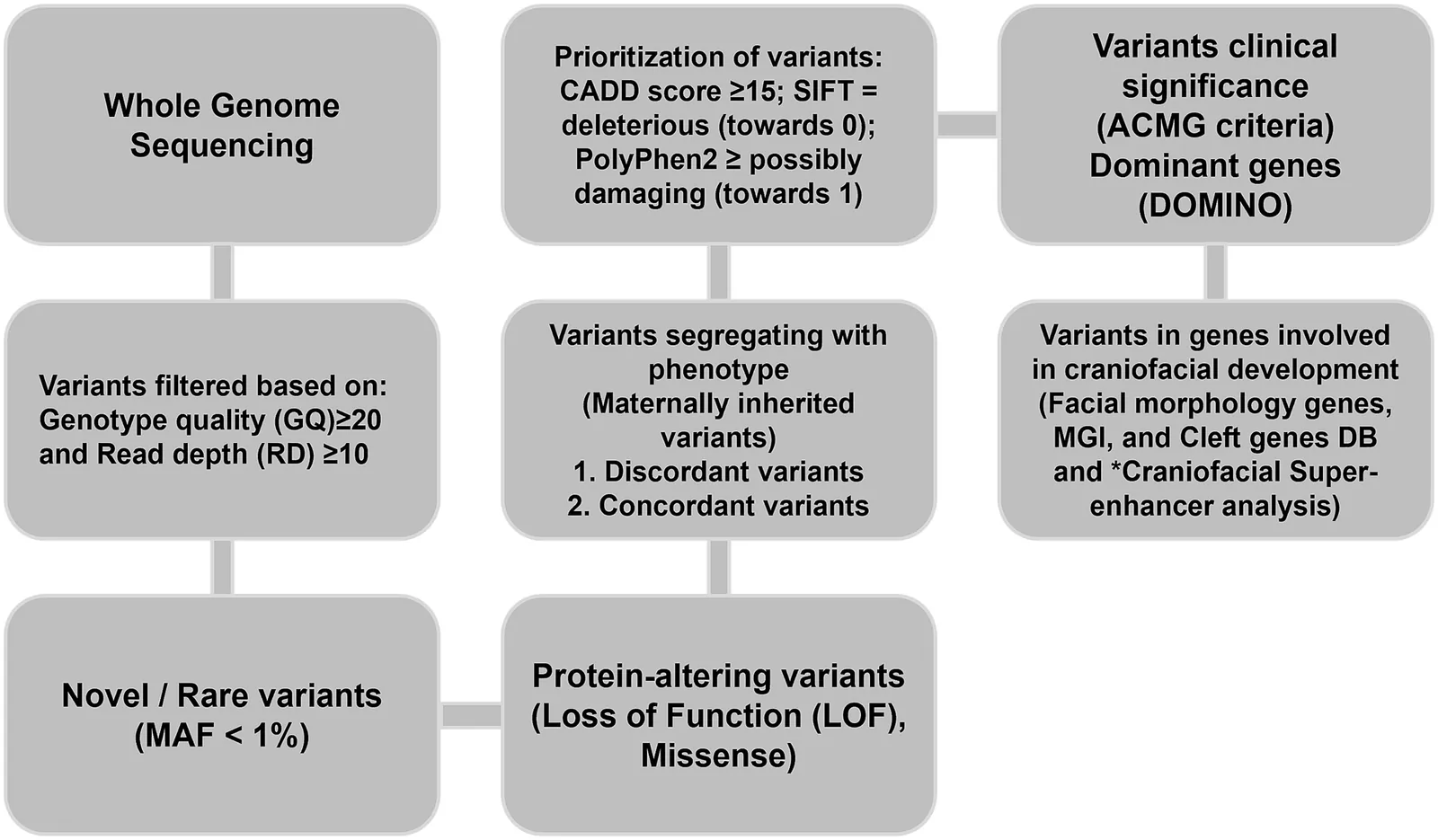
Genomic analytical pipeline used to investigate discordant and concordant pathogenic protein-altering variants in the mirror twins contributing to the risk of cleft.

Craniofacial candidate genes were defined as those with documented roles in craniofacial development based on three curated resources: (1) the Facial Morphology Gene Scan database [17], which catalogues genes associated with normal facial variation; (2) the Mouse Genome Informatics database (MGI; www.informatics.jax.org), queried for genes whose mouse orthologs produce craniofacial or orofacial phenotypes when disrupted; and (3) the CleftGeneDB database of genes associated with cleft phenotypes in animal models (https://bioinfo.uth.edu/CleftGeneDB). The complete list of genes selected based on these criteria is provided in Supplementary Table S2.

### Additional sequencing analysis

We used Sanger sequencing to confirm variant segregation in the family for top candidates from Table 2.

For replication of the *FGF20* finding, we analyzed the *FGF20* genomic region (full transcriptional span plus 1 kb upstream) in whole-genome sequencing data from 1611 multiethnic parent-offspring trios with nonsyndromic CL/P available through the Gabriella Miller Kids First Pediatric Research Program (phs001168, phs001420, phs001997, phs002595, phs003893) [22]. Supplementary Table S3 shows a summary of the public datasets used. Variants were extracted using bcftools and tested for preferential transmission using an allelic transmission disequilibrium test (TDT). A complementary rare variant burden test (MAF < 0.1%) was performed using the same TDT framework with gnomAD allele frequencies.

### *In silico* functional analysis

Following the identification of the main candidate genes, we examined craniofacial regulatory elements signatures and their spatiotemporal expression during embryonic development. Using data from the expanded encyclopedia of DNA elements (ENCODE) (https://www.encodeproject.org), we searched for gene regulatory elements signatures including enhancer-like, promoter-like and CTCF- binding elements. We further analyzed the Human Craniofacial Epigenomics dataset (GSE97752) from the Cotney Lab (https://www.chopcranio.org/human-craniofacial-development) [20] to look for presence of regulatory element signatures*,* revealing craniofacial-specific regulatory elements active during relevant Carnegie stages (CS13-22) [21].

### DNA methylation analysis in twins

Genome-wide DNA methylation profiling was performed using the Illumina EPIC BeadChip array, which evaluates over 850 000 CpG sites. Methylation levels were quantified as β-values (0–1), representing the ratio of methylated to total signal. The EpiDISH package was used for cell-type composition normalization [37]. CpG sites with coefficient of variation > 20% in gender-matched controls were excluded. Differentially methylated positions (DMPs) with absolute Δβ ≥ 5% between twins were selected for downstream analysis. The DNA methylation analysis workflow is illustrated in Fig. 4.

**Figure 4:**
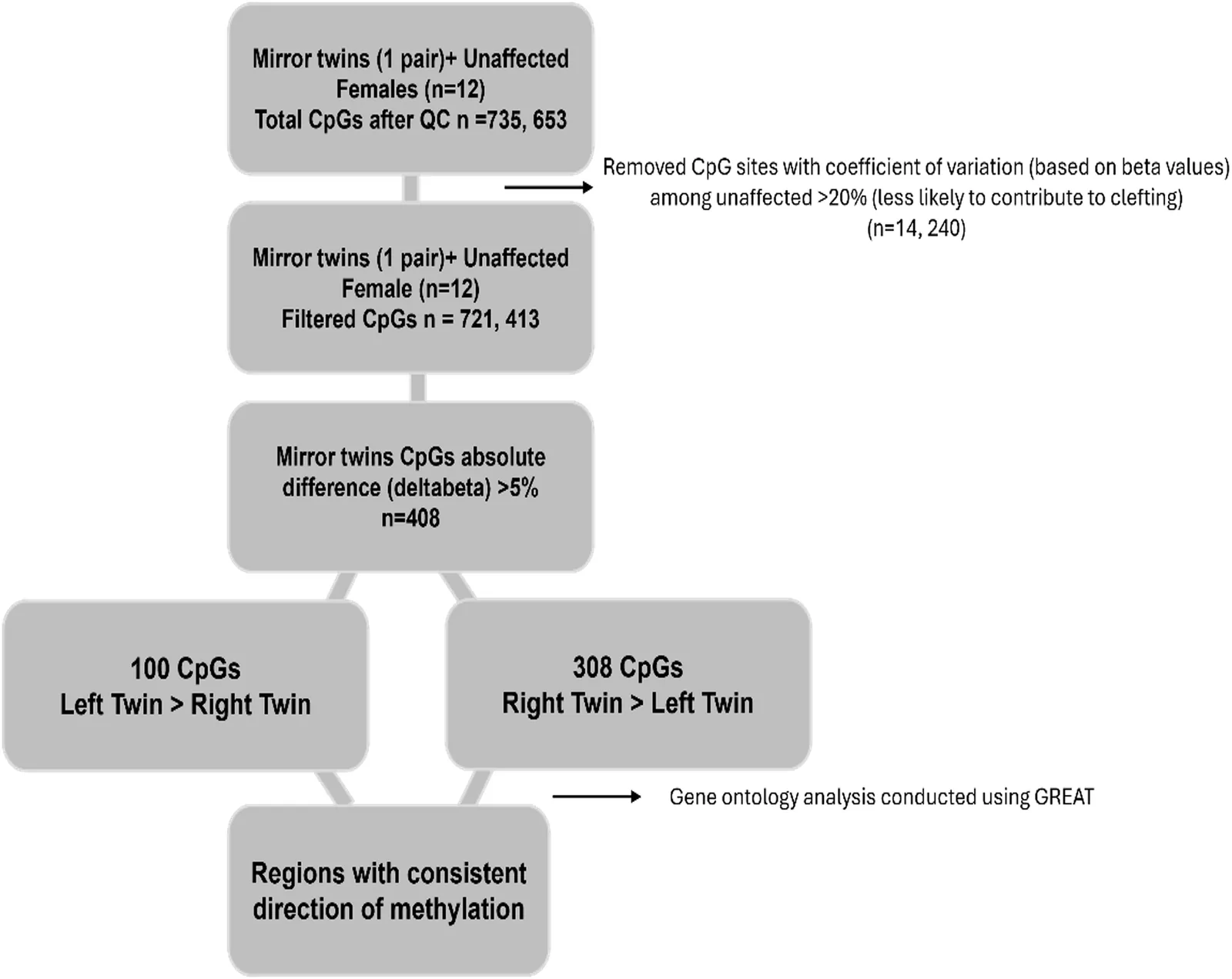
DNA methylome analytical pipeline.

### Methylation replication studies

We performed a ***genome-wide methylation analysis*** (Illumina EPIC array) in 38 individuals with unilateral cleft lip and/or palate, comprising blood samples (right = 9, left = 13) and saliva samples (right = 7, left = 9). This screening identified three genes (*ARID5B, ZFP57*, and *HOOK2)* as the top three differentially methylated candidate loci.

We then followed up with a ***targeted methylation analysis of three priority candidate genes***—*ARID5B* (CpG site cg22368847; n = 377), *ZFP57* (CpG site cg20228636; n = 385), and *HOOK2* (CpG site cg11738485; n = 383)—in individuals with unilateral nonsyndromic CL/P. Each cohort comprised approximately equal proportions of four cleft subtypes: left cleft lip and palate (LCLP), right cleft lip and palate (RCLP), left cleft lip only (LCL), and right cleft lip only (RCL) as shown in Table 5. Critically, analyses were performed separately for blood-derived (*ZFP57*: n = 198; *HOOK2*: n = 196; *ARID5B*: n = 198) and saliva-derived (*ZFP57*: n = 187; *HOOK2*: n = 187; *ARID5B*: n = 179) DNA to account for tissue-specific methylation patterns [30]. To compare methylation levels between two groups (e.g. left vs. right clefts), the normality of each group’s distribution was assessed using the Shapiro–Wilk test. The nonparametric Mann–Whitney U test (equivalent to the Wilcoxon rank-sum test) was applied for all two-group comparisons, as the majority of subgroups exhibited significant departures from normality. For comparisons across four cleft subtypes (LCLP, RCLP, LCL, RCL), the Kruskal-Wallis test was used. When the Kruskal-Wallis test indicated a significant difference, pairwise post-hoc comparisons were conducted using Mann–Whitney U tests with Bonferroni correction applied across all six pairwise comparisons (adjusted significance threshold α = 0.0083). All analyses were conducted using the SAS® System version 9.4 (SAS Institute Inc., Cary, NC, USA). Statistical significance was set at *P* < .05.

**Table 5:** Patients distributions and characteristics per candidate gene for the targeted replication cohort.

	Source n (%)		Sex n (%)		Race n (%)		Cleft Type n (%)				Laterality n (%)	
Gene	Saliva	Blood	Male	Female	White	Non-White	LCLP	RCLP	LCL	RCL	Left	Right
*ZFP57*	187 (48.6)	198 (51.4)	203 (52.7)	182 (47.3)	91 (23.6)	294 (76.4)	99 (25.7)	96 (24.9)	96 (24.9)	94 (24.5)	195 (50.6)	190 (49.4)
*HOOK2*	187 (48.8)	196 (51.2)	204 (53.3)	179 (46.7)	89 (23.2)	297 (76.3)	96 (25.1)	94 (24.5)	94 (24.5)	99 (25.9)	190 (49.6)	193 (50.4)
*ARID5B*	179 (47.5)	198 (52.5)	201 (53.3)	176 (46.7)	90 (23.9)	287 (76.1)	94 (24.9)	92 (24.4)	97 (25.8)	94 (24.9)	191 (50.7)	186 (49.3)

LCL-left cleft lip; RCL-right cleft lip; LCLP-left cleft lip and palate; RCLP-right cleft lip and palate

To distinguish laterality effects from cleft-extent effects, two complementary analyses were performed for each gene: (1) a direct left vs. right comparison pooling all cleft subtypes (LCLP+LCL vs. RCLP+RCL) and (2) a four-subtype Kruskal-Wallis test followed by post-hoc pairwise comparisons to identify which specific subtype contrasts drove any significant omnibus result. This design allows laterality-specific signals (e.g. LCL vs. RCL; LCLP vs. RCLP) to be distinguished from cleft-extent signals (e.g. CLP subtypes vs. CL subtypes) within the same analytical framework.

## Supplementary Material

Supplementary_material_ddag088

## Data Availability

The data supporting the findings of this study are available from the corresponding author upon reasonable request.
